# Feasibility of a disposable canister-free negative-pressure wound therapy (NPWT) device for treating open wounds in horses

**DOI:** 10.1186/s12917-019-1829-5

**Published:** 2019-03-06

**Authors:** Louis Kamus, Marie Rameau, Christine Theoret

**Affiliations:** Département de biomédecine vétérinaire, Université de Montréal, 3200 rue Sicotte, St-Hyacinthe, QC J2S 2M2 Canada

**Keywords:** Horse, Wound, Negative pressure wound therapy, Feasibility

## Abstract

**Background:**

Wounds are among the most common medical conditions affecting horses and have a major economic impact on the horse industry. Wound healing in horses is distinct to that documented in other species, and often results in delayed healing and extensive scarring, with compromised functional and aesthetic outcomes. To date, there is no conventional method objectively proven to accelerate healing or to successfully prevent complications associated with second intention healing. Several effects of Negative Pressure Wound Therapy (NPWT) may be particularly useful to the management of wounds in horses. However, cumbersome designs of classic NPWT devices render them unsuitable for equine practice. A new lightweight, portable and disposable unit of NPWT (PICO®), should facilitate the use of this modality by equine practitioners. The aim of this study was to evaluate the feasibility of using this canister-free system to treat experimental open wounds in horses.

**Results:**

No difficulties were encountered with the application or maintenance of the PICO® system during the ex vivo experiment or during the preliminary in vivo experiment conducted on intact skin. All horses readily tolerated the PICO® but difficulties with adhesion and seal prevented the completion of the experimental wound study despite the use of many adjunctive adhesives.

**Conclusion:**

The current PICO® dressing design is not suitable to be used as a dressing for open wounds in horses though the device is well tolerated by equine patients. A dressing with a wider adhesive edge, a superior adhesive and a more flexible pad would likely be better adapted to enable its future use in equine practice.

**Electronic supplementary material:**

The online version of this article (10.1186/s12917-019-1829-5) contains supplementary material, which is available to authorized users.

## Background

Wounds have a major economic impact on the horse industry [1–5]. A large study by the United States Department of Agriculture found that wound/trauma/injury was the most common medical condition affecting horses [4]. It was the leading cause of death in horses less than 1 year old, accounting for 27.8% of deaths, and was the second leading cause of mortality after old age [4].

The wound healing process in horses is distinct to that documented in other species, and often results in delayed healing and extensive scarring, with compromised functional and aesthetic outcomes [6]. Second intention healing in horses is often fraught with complications. These complications mostly affect wounds on the distal limb where healing is characterized by a weak acute inflammatory response [7] followed by chronic inflammation, commonly leading to the development of Exuberant Granulation Tissue (EGT) [8] and a subsequent delay in epithelialization, contraction and wound closure. Excessive environmental contamination in the horse’s environment and the proximity of the wound to the ground are considered contributing factors.

To date, there is no conventional method objectively proven to accelerate healing or to successfully prevent EGT. Consequently, there is an unmet need to investigate adjunctive treatment modalities shown to support wound healing in human medicine. Negative pressure wound therapy (NPWT) has become the first line of treatment for surgical wounds, skin grafts, chronic wounds as well as acute accidental contaminated wounds in man [9–12]. Several notable effects of NPWT may be particularly useful to the management of accidental wounds in horses. For example, the prevention of wound retraction, decrease in wound size, reduction of wound bioburden and wound edema, and early onset of fibroplasia should be beneficial to wounds healing by second intention or for wounds being prepared for delayed primary closure. NPWT is shown to increase blood flow to the wound bed and formation of granulation tissue and to accelerate removal of wound exudate [13–15]. It is also stated that NPWT induces an anti-inflammatory profile in the wound bed combined with an effect on remodeling of the extracellular matrix [16]. However, the cumbersome design of classic NPWT devices renders them inconvenient for equine practice as these devices are often heavy and secured with difficulty on a horse. A new minimalist lightweight, portable (canister-free) and disposable unit of NPWT, PICO® from Smith & Nephew (Lachine, Canada), is expected to facilitate the use of this modality by equine practitioners.

A few case reports, as well as retrospective and prospective studies in cats [17, 18] and dogs [19–24] have already described the use of negative pressure to treat different types of wounds: traumatic wounds, burn injuries, infected surgical incision and skin grafts. In addition to occurring earlier, the granulation tissue filling a wound treated by NPWT in dogs has been noted to be smoother, not exuberant, and histologically demonstrates well-organized collagen fibers [20], suggesting a decreased likelihood for NPWT-treated wounds to develop EGT in dogs. However, the mechanisms of action and effect on wound healing in horses need to be clarified as the literature is more scarce in this species. One case report in a horse describes the use of a non-portable NPWT device, with a canister, on an extensive open wound of the neck and concludes that second intention healing is facilitated and accelerated by NPWT [25]. A second case report describes the use of NPWT in three horses with an open and infected subcutaneous olecranon bursa [26]. The successful use of NPWT on punch grafts in a horse has also been reported [27]. The antibacterial effect of NPWT using 3 different foams in an equine ex vivo wound model was also investigated [28]. Controlled experimental and clinical studies are needed in horses to assess if NPWT increases the rate of wound healing through acceleration of its different phases, has an effect on the production of EGT and improves the final outcome of healing wounds.

Our study aimed to assess the feasibility of using the PICO® vacuum unit to administer continuous negative pressure to experimental open wounds in horses. We hypothesized that a vacuum can be successfully maintained with no signs of pain or discomfort to experimental open wounds in horses and that voluntary movement and manipulation of the patients would not preclude maintenance of the vacuum.

## Results

### Preliminary ex vivo experiment

Vacuum was achieved upon first application in all cadaveric limbs, as confirmed by the green light and wrinkling of the dressing pad occurring within 30s. The vacuum was successfully maintained on each limb for 24 h.

### Preliminary in vivo experiment

Vacuum was achieved upon first application in both mares, as confirmed by the green light and wrinkling of the dressing pad occurring within 30s. The vacuum was successfully maintained, with the mares under observation, for a pilot period of one hour. The mares showed no signs of pain or discomfort (as measured by increased heart and respiratory rates, and observation of behavioral patterns). Voluntary movement and manipulation of the mares did not preclude maintenance of vacuum during the experiment.

### Experimental wound study

Since no difficulties were encountered with the application or maintenance of the PICO® system during the ex vivo experiment or during the preliminary experiment conducted on intact skin in live horses, it was decided to proceed with the experimental wound study.

Two groups of two mares started the experiment at a 24 h interval from one another. A slight erythema and subcutaneous swelling was noted on the thoracic wall of one mare following application of the hair removal cream prior to wounding. This complication resolved in 48 h and was not observed in the other mares. Although no significant hemorrhage occurred during the first 24 h after wounding, subcutaneous edema developed around thoracic wounds, particularly along the distal border where tunneling was performed.

**Fig. 1:**
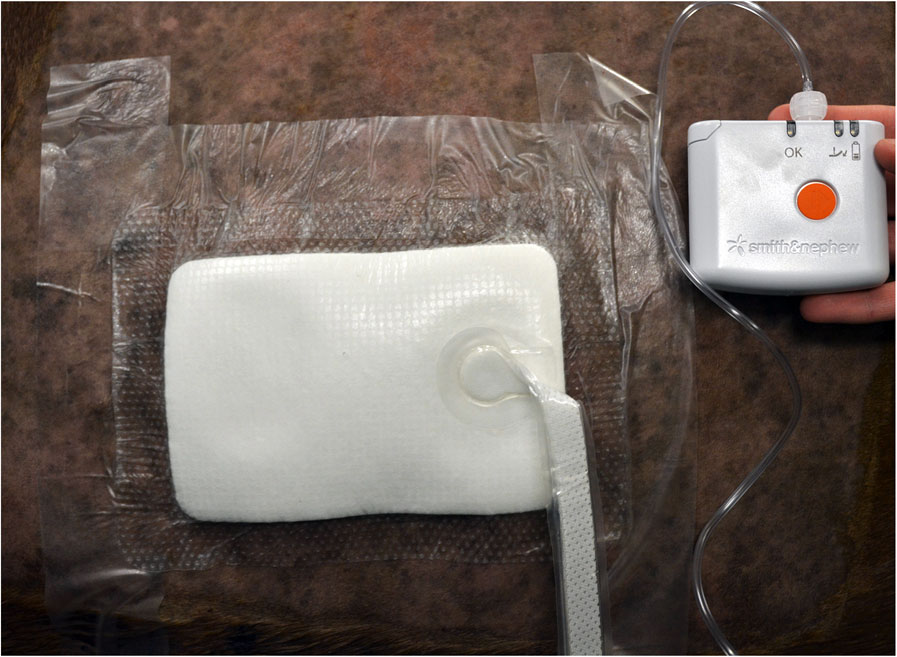
Test application of the PICO® dressing to thoracic wounds in association with polyurethane adhesive film (OPSITE®, Smith & Nephew) strips placed over the dressing edges to improve adhesion

Difficulties were encountered during the first application of the PICO® dressing, at both anatomic locations, in the first group of two mares. In spite of a thorough initial cleansing with Chlorhexidine gluconate 4% soap (HibiScrub, Mölnlycke Health Care, Oakville, Canada) followed by careful drying of the skin surrounding the wound margins, the silicone adhesive layer of the PICO® dressing failed to adhere to the skin. The addition of an adhesive polyurethane film (OPSITE®, Smith & Nephew) over the dressing’s edges did not improve adhesion as this film also failed to stick to the skin (Fig. 1). As a consequence of the ineffective seal, the vacuum was not achieved at this stage of the experiment. In an effort to preclude the possibly negative effect of the subcutaneous edema having developed around the thoracic wounds, the PICO® dressing was applied immediately following wound creation, at both anatomic locations, in the second group of two horses. In this case, an effective vacuum was achieved in the thoracic area but successfully maintained for only 15 to 20 min. A satisfactory vacuum was not obtained on the limb wounds in any of the four mares. The PICO® hardware was well tolerated by all the mares, for a 24 h period in their stalls.

In view of these unexpected difficulties, an additional set of tests was then undertaken on a separate, unwounded, mare to determine the best configuration to successfully apply the PICO® dressing. Various elements and conditions were tested in an effort to improve the dressing adhesion and vacuum pump efficacy. The initial skin preparation was revised since, during the experimental wound study, two mares were observed to sweat profusely following sedation, which may have reduced the adhesive properties of the PICO® dressing. Consequently, an antiperspirant spray (MEN REXONA Adventure®, Unilever Canada Inc., Toronto, Canada) was applied locally following skin preparation, though this did not improve adhesion of the dressing. A liquid dressing (SKIN-PREP®, Smith & Nephew) was also used to prepare the skin following cleansing and before dressing application, but this also failed to improve adhesion of the PICO® dressing to the horse’s skin. The adhesion of the dressing on the distal limb was tested with and without prior treatment with hair removal cream to assess the cream’s and the hair’s potential effect on the adhesive properties of the PICO® dressing. Although good dressing adhesion was obtained in both cases, the pump was unable to maintain an effective vacuum. At the suggestion of the manufacturer, the vertical depressions between the second and fourth metacarpal bone and the suspensory ligament were filled with 4 × 4 woven gauze to eliminate dead space, however, this did not help to achieve an effective vacuum.

Various adjunctive adhesives were also tested at the periphery of the thoracic wounds of the four mares in which application of the PICO® device failed: cyanoacrylate glue (Krazy Glue®, Krazy glue Inc., High Point, NC), spray adhesive (Mueller Tuffner Pre-tape spray, Mueller sport medicine Inc., Prairie du Sac WI), gel adhesive (RENASYS® Adhesive *gel patch*, Smith & Nephew) and silicone gel patches (CicaCare®, Smith & Nephew) all failed to improve adhesion sufficiently for vacuum to be maintained. Other elements were superimposed on the edges of the PICO® dressing: a spray dressing (OPSITE SPRAY®, Smith & Nephew), a liquid dressing (NewSkin Liquid bandage, Moberg Pharma North America LLC, Cedar Knolls, NJ) and an adhesive elastic band (TENSOPLAST®, BSN Medical Canada, Laval, Canada); these also failed to enhance adhesion of the PICO® dressing.

**Fig. 2:**
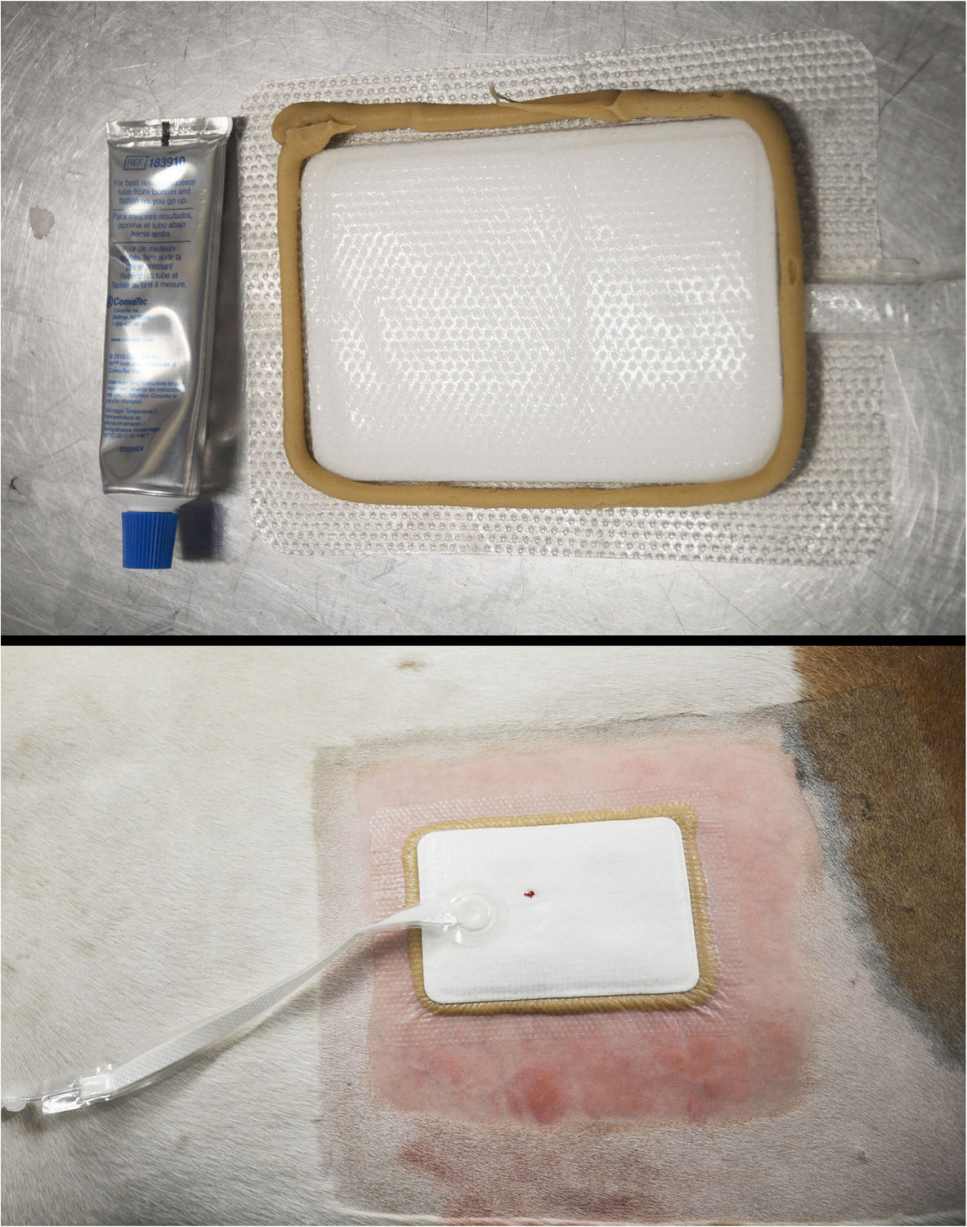
Application of a stoma paste (Stomahesive®, ConvaTec, Dorval, Canada) to the edges of the PICO® dressing in an effort to improve adhesiveness and enable effective vacuum to be achieved

**Fig. 3:**
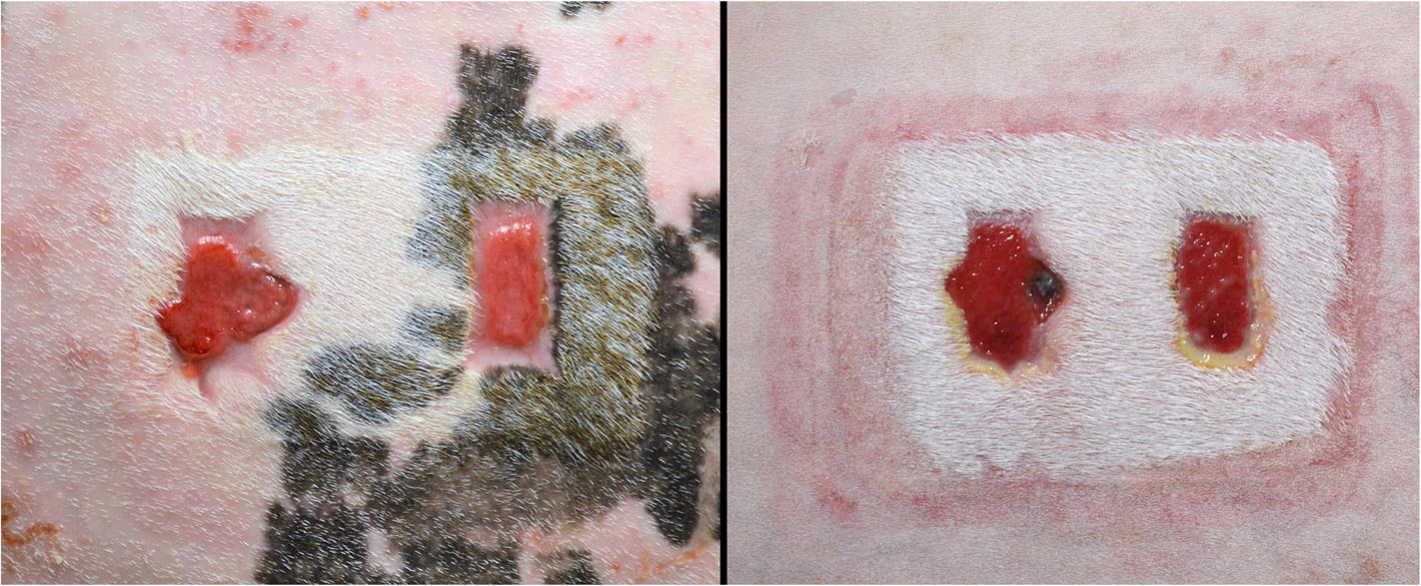
Cutaneous irritations and subcutaneous edema caused by stoma paste. Irregularities seen at the wound edges are the result of wound harvesting during the experiment intended for histological and molecular analyses

The application of a stoma paste (Stomahesive®, ConvaTec, Dorval, Canada) to the edges of the PICO® dressing generated variable results (Fig. 2). Adhesion of the dressing was sufficient to allow an effective and stable vacuum to be achieved and maintained for 24 to 48 h on the thoracic wounds of three mares and on the thoracic skin of the unwounded mare. The mares received a 7-day NPWT with frequent dressing change; indeed, focal liquefaction of the stoma paste was consistently observed 24 h after application and precluded vacuum maintenance, obliging dressing change 3 to 4 times a day. Moderate cutaneous irritations and subcutaneous edema were observed on all mares at the first dressing change (Fig. 3). In these mares, the PICO® NPWT system was well tolerated by three individuals for the week-long trial, whereas one individual required the installation of a shoulder protection (usually used to prevent blanket rubbing over the shoulder) to shield the vacuum pump. No behavioral patterns indicating pain or discomfort, nor changes in heart rate or respiratory rates associated with use of the device were observed during the daily clinical examination for the week-long trial.

**Fig. 4:**
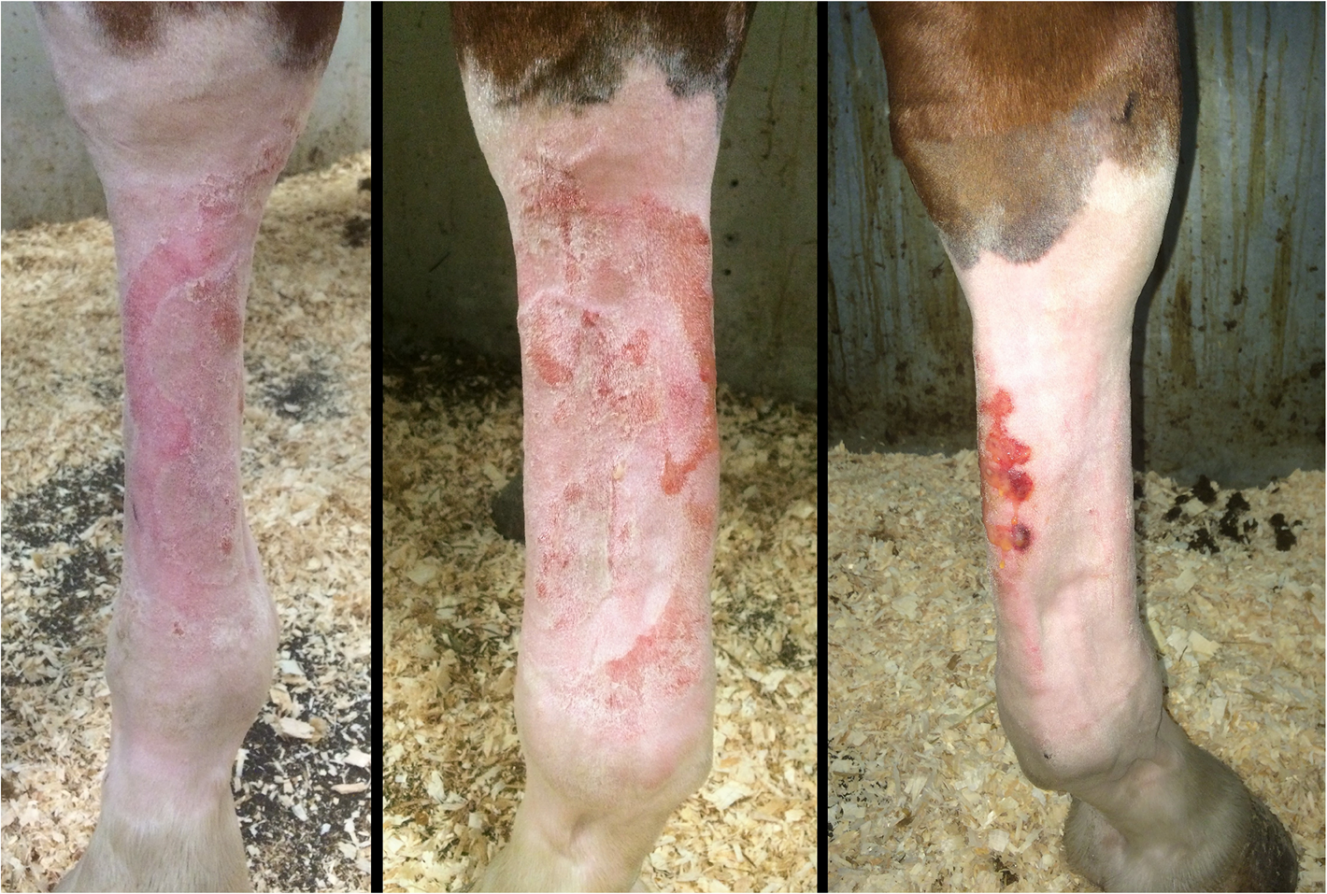
Severe cutaneous irritations caused by the stoma paste 4 days after application on the distal limb of an unwounded mare. Irritations follow the edges of the PICO Multi-site® dressing

A special dressing designed for complex anatomical areas (PICO Multi-site®, Smith & Nephew) was tested, with the stoma paste, on two distal forelimbs: one on a mare of the experimental wound group and one on the mare without wounds. Moreover, a breathable bandage consisting of cotton undercast padding (Webril®, Covidien-Medtronic), stretch bandage (Curity®, Covidien-Medtronic) and bandaging tape (VetRap®, 3 M Canada, London, Canada) was applied over the PICO® dressing. This design enabled the achievement and maintenance of a vacuum for 4 consecutive days. However, cutaneous irritations were noted in both cases after dressing removal at 4 days: the mare with experimental wounds had minor irritations whereas the unwounded mare developed severe cutaneous irritations associated with cellulitis (Fig. 4). These irritations were treated with daily hydrotherapy, bandages and a course of anti-inflammatory drug (phenylbutazone, 2.2 mg/Kg, BID) for 3 days.

Once the NPWT trial was abandoned, 2 weeks after the wound surgery, all wounds were left to heal by second intention (Additional file 1: Figure S1). Limb wounds took longer to heal than did body wounds: average time to full healing for limb wounds was 83 days (SD 2.58 days) and that for body wounds was 62.5 days (SD 2.52 days).

## Discussion

Our experimental wound study aimed to assess the feasibility of using a small, portable unit (PICO®) to administer negative pressure to open wounds in horses. The highly-evaporative dressing (with a purported capacity of 300 mL of wound exudate) of the PICO® system obviates the need for a canister to manage exudate, rendering the system both disposable and ultra-portable. This lightweight design is appealing for veterinary wound management since some patients cannot be confined to a cage or a stall for the duration of therapy and do not tolerate a more heavy design of vacuum pump. A recent retrospective study described clinical experience with the PICO® system to successfully manage free skin grafts on the limbs of seven dogs [21]. In our study, the majority of horses readily tolerated the PICO® system during 7 days since it allowed normal mobility. Supplementary device protection was required for only one mare, demonstrating that the design of the PICO® vacuum pump is well suited for use in horses.

Difficulties were however encountered with the dressing component of the PICO® system, although preliminary testing on unwounded hairless limb/thoracic skin and during the ex vivo experiment did not foretell these problems, encouraging our team to proceed with the experimental wound study. The good results obtained on intact skin were unfortunately not reproducible in our experimental wound study and might indicate that inflammation and edema caused by wounding and tunneling of the thoracic wounds may have contributed to the problems encountered. A satisfactory vacuum could not be obtained for limb wounds when using only the PICO® dressing, possibly because the vertical cleft in the region distal to the splint bones created an air pocket that may have counteracted the force of the pump. According to the manufacturer, the primary contact silicone layer is designed to minimize pain upon removal and to adapt to skin irregularities and scattered small hairs on the patient’s skin, however, compared to humans, horses have a very high pilosity and thicker individual hairs, especially on the distal limb, which may have precluded the purported adaptability of the silicone layer. No major difficulties in adhesion of the PICO dressings were reported in a recent study in dogs [21]. We are not aware of studies directly comparing horse and dog skin that could help explain the adhesion problems encountered in our study. However, horse skin characteristics are known to differ from those of other species [29]. Horses have thicker skin [30] especially over the dorsal and lateral surfaces of limbs compared to the rest of the body [31, 32]. Horse skin is well endowed with numerous sweat glands (apocrine glands), physiologic control of sweating is different, and horses sweat more and in different patterns than most other species. Also, apart from being strongly alkaline, horse’s sweat contains a high concentration of a glycoprotein called latherin, which is believed to act as a wetting agent to facilitate heat dissipation through the oily coat of horses [33]. Moreover, surface lipids of horse skin have been studied and are different from those of humans. They include a high percentage of lactones, sterol esters, cholesterol and wax diesters [34, 35]. As yet unestablished, we suspect that these characteristics may have led to the adhesion difficulties encountered in our study especially in light of the dressing adhering well to wounded cadaveric limbs of horse in the preliminary ex vivo experiment. Interestingly, during the trial, a PICO® dressing that failed to adhere to horse skin was then applied successfully to the intact skin of one of the researchers, confirming our impression that the current design of the PICO® dressing, while perfectly suited to human patients, is not optimized for horse skin. To enhance the adhesion capacity of the primary contact silicone layer, different adjunctive products and approaches were tested, but failed. Although it may be tempting to use saran wrap plastic or an Ioban antimicrobial incise drape, the use of any occluding material is precluded by the design of the PICO® dressing pad’s external-most layer, which ensures transpiration of exudate. The best functional outcome was obtained with a stoma paste applied to the edges of the PICO® dressing pad, enabling a better contact with the thoracic skin and facilitating the work of the vacuum pump. Similar results were achieved on the distal limb with a combination of stoma paste, a more flexible PICO® dressing (PICO Multi-Site®, Smith & Nephew) and a traditional half-limb bandage without the secondary layer. However, although the PICO® system functioned with these modifications, complications including moderate to severe cutaneous irritations and cellulitis preclude the use of stoma paste to ensure adequate adhesion of the currently available PICO® dressing in horses.

## Conclusion

In conclusion, our experience suggests that the current design of the PICO® NPWT dressing is not suitable for use in the treatment of open wounds in horses though the device is well tolerated by equine patients. A dressing with a wider adhesive edge, a superior adhesive and a more flexible pad would likely be better adapted to enable its future use in equine practice.

Although our study did not provide the anticipated outcome, we are convinced that communication of the experience we gleaned will help prevent duplication of research effort and better direct future efforts and resources.

## Methods

### Negative-pressure wound therapy device

The simplified NPWT device (PICO®, Smith & Nephew) consists of a vacuum unit and a four-layered dressing connected through tubing. The vacuum unit (8.5 × 8.5 × 2 .5cm in size and weight < 120 g) is powered with two AA lithium batteries and is programmed to deliver a continuous negative pressure of − 80 mmHg [36]. A green light on the pump illuminates to indicate that an airtight seal has been obtained, while an orange indicator light and a lack of wrinkling of the dressing are indicative of leaks. The patented four-layered dressing consists of a primary contact silicone layer, an airlock layer that allows even distribution of negative pressure, an absorbent layer that moves exudate away from the wound and an outer vapor transmission layer that allows one-way transpiration of exudate. The manufacturer suggests applying bands of adhesive polyurethane film (OPSITE®, Smith & Nephew) over the silicone edges of the dressing to improve the seal between the dressing and the skin. The dressing does not normally need daily changing (unless the 300 mL capacity of the dressing has been reached due to a large amount of wound exudate).

### Preliminary ex vivo experiment

**Fig. 5:**
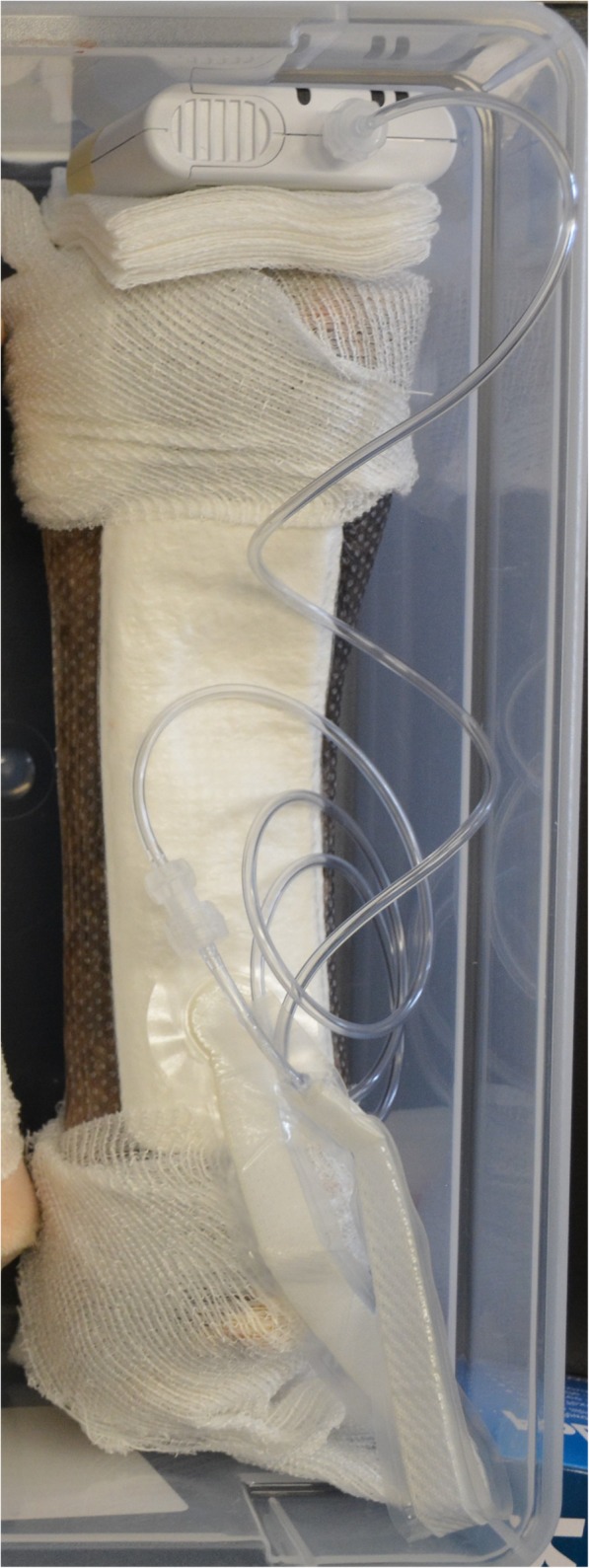
Ex vivo experiment on equine cadaveric limbs

The PICO® system was first tested on cadaveric limbs to verify the ability to secure the PICO® dressing to (unwounded) equine limb skin and to successfully maintain a vacuum during the experiment. Four equine thoracic forelimbs were obtained from a local slaughter-house on the day of testing. Limbs were disarticulated at the middle carpal joint and at the metacarpophalangeal joint, to keep only the metacarpal area, which was clipped, scrubbed with 2% chlorhexidine gluconate soap and subjected to depilation with hair removal cream (Veet®, Reckitt Benckiser, Berkshire, UK). After an aseptic preparation including a 5-min 4% chlorhexidine gluconate scrub followed by a 70% alcohol rinse, a rectangular (2X3cm) full-thickness skin excision was surgically created on the dorsolateral surface of the third metacarpal bone. A PICO® dressing (15x20cm) was applied to the dorsolateral surface of the third metacarpal bone (Fig. 5). Bands of adhesive polyurethane film (OPSITE®, Smith & Nephew) were applied at the junction between the silicone edges of the dressing and the skin. The pump was then connected to the evacuation tubing exiting the dressing. All limbs were monitored every 6 h during 24 h for vacuum leakage and maintenance of the dressing.

### Preliminary in vivo experiment

A pilot study was performed on two mares from the teaching herd of the veterinary faculty. The primary aim of this part of the study was to determine patient tolerance of the device.

**Fig. 6:**
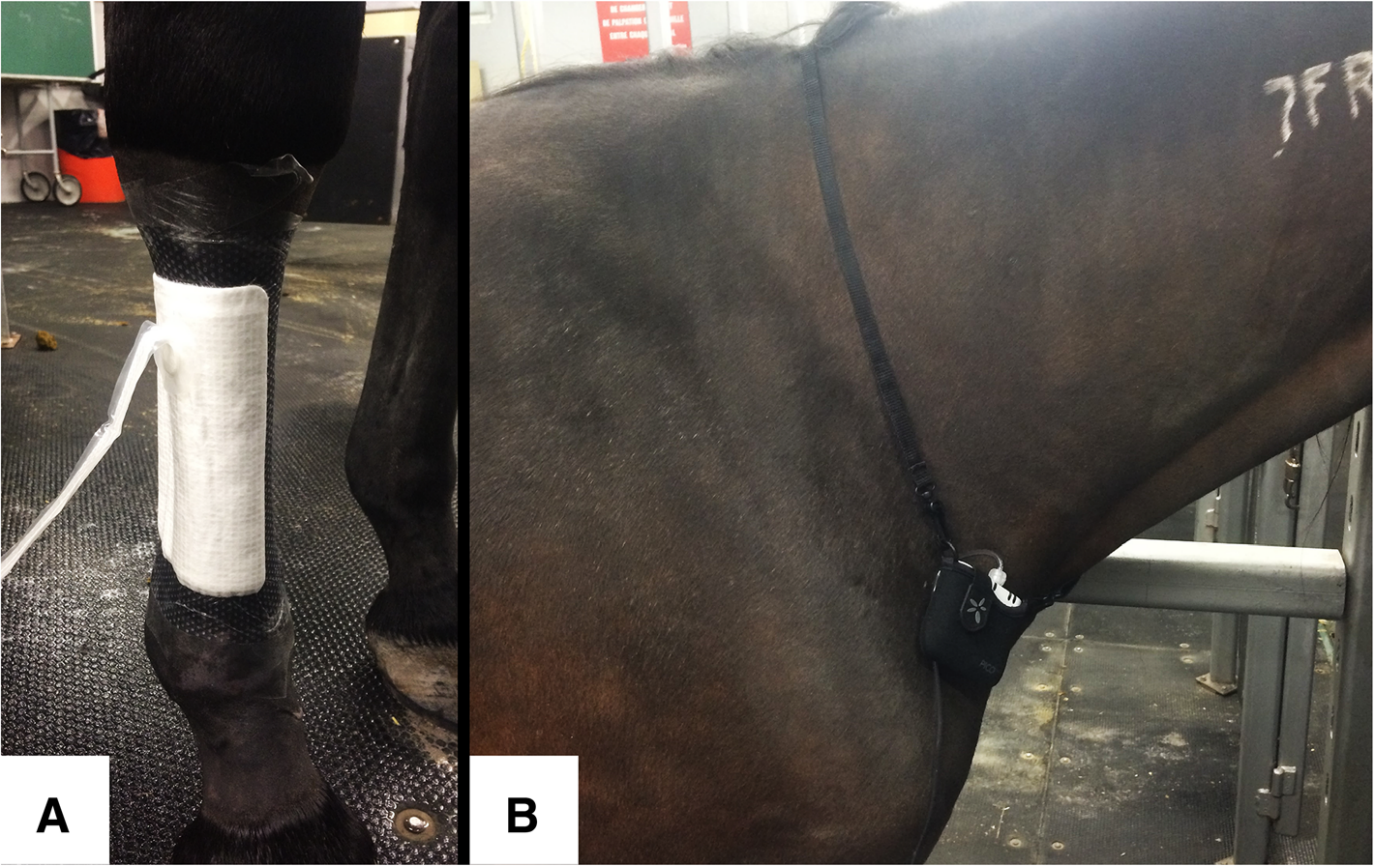
Preliminary in vivo experiment. **a**, PICO® dressing (15x20cm) in place, over the dorso-lateral and lateral surfaces of the third metacarpal bone of an unwounded horse. **b**, PICO® vacuum pump in the pouch supplied by the manufacturer, held in place around the neck of a horse using the strap provided by the manufacturer

*Limb:* The distal extremity of the right forelimb of one mare was clipped and scrubbed with 2% chlorhexidine gluconate soap. Hair removal cream (Veet®, Reckitt Benckiser) was used on the canon, fetlock and pastern according to manufacturer instructions. A PICO® dressing (15x20cm) was applied to the dorsolateral surface of the cannon with the suction port positioned proximally and the evacuation tubing coursing towards the mare’s trunk. Bands of adhesive polyurethane film (OPSITE®, Smith & Nephew) were applied at the junction between the silicone edges of the dressing and the skin. The pump was then connected to the evacuation tubing exiting the dressing and attached to the horse’s neck using the pouch and strap supplied with the device (Fig. 6).

*Thorax:* The same skin preparation was used on the right hemithorax of a second teaching mare and the PICO® system was applied using a 10x20cm dressing. The pump was then connected to the dressing and attached to the horse’s neck using the pouch and strap supplied with the device.

Both mares were monitored during one hour for vacuum leakage, maintenance of the dressing and for tolerance to the system. Pain or discomfort associated with the device was assessed by measuring the heart and respiratory rates. Any behavioral changes (e.g. flanking, pawning) associated with use of the device were also noted.

### Experimental wound study

This study was experimental, prospective, controlled and randomized with each subject being its own control.

**Fig. 7:**
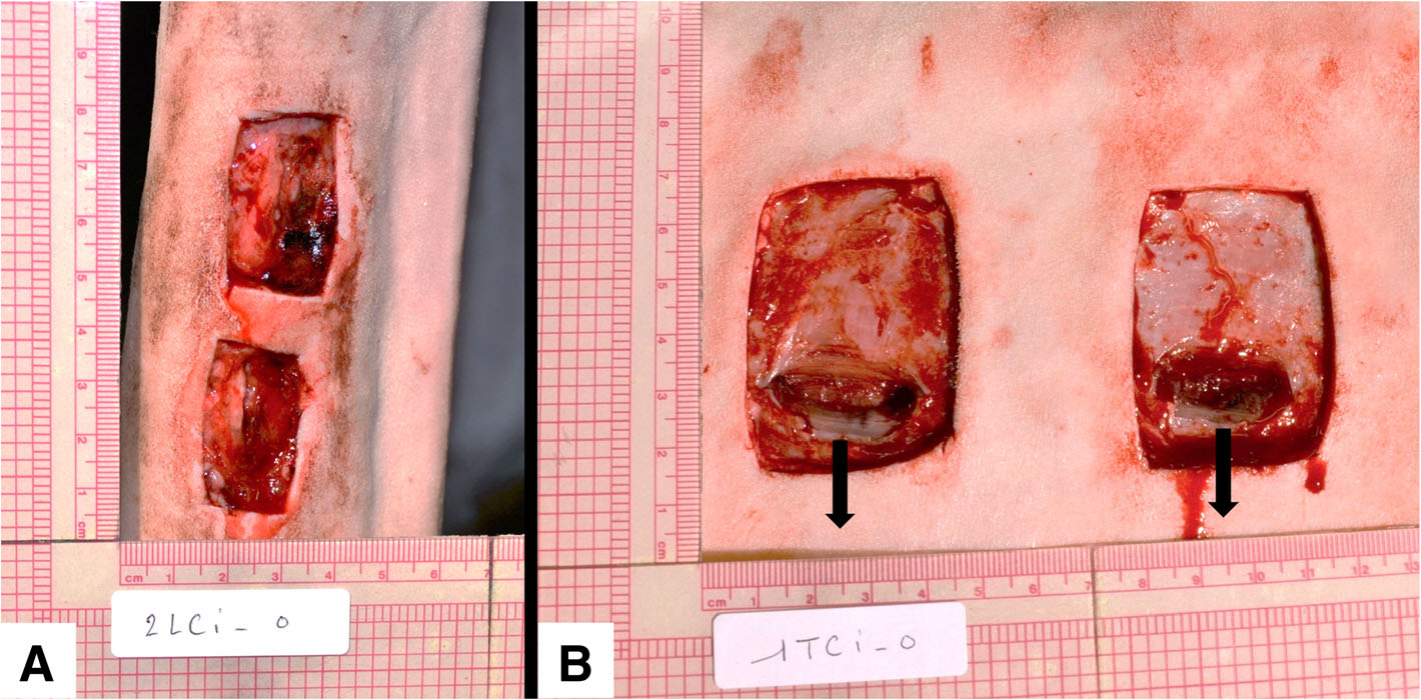
Wound model for in vivo experiment. **a**, distal limb. **b**, thorax, showing muscular tunneling along the distal border (arrow)

Five healthy horses (5 mares) with no evidence of dermatological disease, wounds or scarring on distal forelimbs or lateral thoracic wall were included. All horses were bought from a private horse dealer. Mean age of the horses was 9 years (range 5–15) with a mean weight of 494 Kg (range 450–550). Breeds included Quarter Horse (2), Thoroughbred (1) and mixed breed (2). Horses were dewormed (Moxidectin and Praziquantel, Quest® Plus, Zoetis) and vaccinated (Vetera® Gold^XP^, Boehringer Ingelheim, and IMRAB® Large animal, Merial Inc.) and allowed an acclimation period of 2 weeks following purchase. All horses were fed with ad libitum hay and water. All mares (4) except the Thoroughbred mare were used for the experimental wound study. The Thoroughbred mare was later use in the study for dressing adhesion testing. After the completion of the study, all horses were introduced in the research herd of another research department. One hemithorax and one distal forelimb from the same side were randomly choosen, clipped, scrubbed with 2% chlorhexidine gluconate soap and depilated with hair removal cream (Veet®, Reckitt Benckiser). Horses were sedated with detomidine hydrochloride (0.01 mg/Kg, intravenously [IV]) and butorphanol tartrate (0.04 mg/Kg, IV). No antibiotic or anti-inflammatory treatments were given prior to or after wounding. Local anesthesia was performed using 2% lidocaine hydrochloride: an inverted L-block was used just below the carpus (15 mL, 5 cm above the surgical site) to desensitize the dorsolateral surface of third metacarpal bone while an inverted L-block performed above the wounding area (20 mL, 5 cm away from the surgical site) desensitized the hemithorax. Based on our previously established limb/body wound model [37–39], full-thickness skin wounds were created on the distal extremity of the thoracic limbs (two, 6cm^2^ wounds per limb) and on the lateral mid-thoracic wall (two, 15cm^2^ wounds per hemithorax) (Fig. 7). In the thoracic wounds a 5 cm tunneling of the distal muscular plane of each wound was realized with Metzenbaum scisors in order to create a pocket where exudate might accumulate and thus simulate a clinical situation. All wounds were left to heal by second intention. All wounds on the limb/body were assigned to the NPWT for 7 days, after which wounds would be left without treatment until complete healing.

*NPWT*: The protocol was established in consultation with the manufacturer (Smith & Nephew) and based on a review by the British Association of Plastic, Reconstructive and Aesthetic Surgeons [40]. The limb was bandaged for the first 24 h postoperatively, to control hemorrhage. Wounds on the thoracic wall were left unbandaged during this time. After 24 h, the limbs were unbandaged and the PICO® dressings were applied to wounds in both anatomical locations. Wounds on the limb and thoracic wall were supposed to receive a 7-day treatment using a 15 × 20 cm dressing connected to the PICO® pump.

All mares were monitored four times a day for vacuum leakage, maintenance of the dressing and for tolerance to the system. Pain or discomfort associated with the wound was assessed with daily physical and lameness examination. Any behavioral changes (e.g. flanking, pawning) associated with the device or the wound were also noted.

Under sedation and local anesthesia, full-thickness wound margin samples were harvested from one wound per site (treated body; control body; treated limb; control limb) at days 0 (time of wound creation), 1, 3, 8, and 17, and at the time of full healing. These samples were intended for another wound healing study.

## Additional file

Additional file 1: **Figure S1.** Healing of experimentally induced wounds at the different body sites in a representative horse. A: 24 h, B: 7 days, C: 14 days, D: 21 days, E: cicatrix. (TIF 2334 kb)

## Abbreviations

EGT: Equine granulation tissue
IV: Intravenously
NPWT: Negative pressure wound therapy
