# Supplementary figures and images for: Feasibility of a disposable canister-free negative-pressure wound therapy (NPWT) device for treating open wounds in horses
Source: BMC Vet Res. 2019 Mar 6;15:78. doi: 10.1186/s12917-019-1829-5 (PMC6404353; doi:10.1186/s12917-019-1829-5)

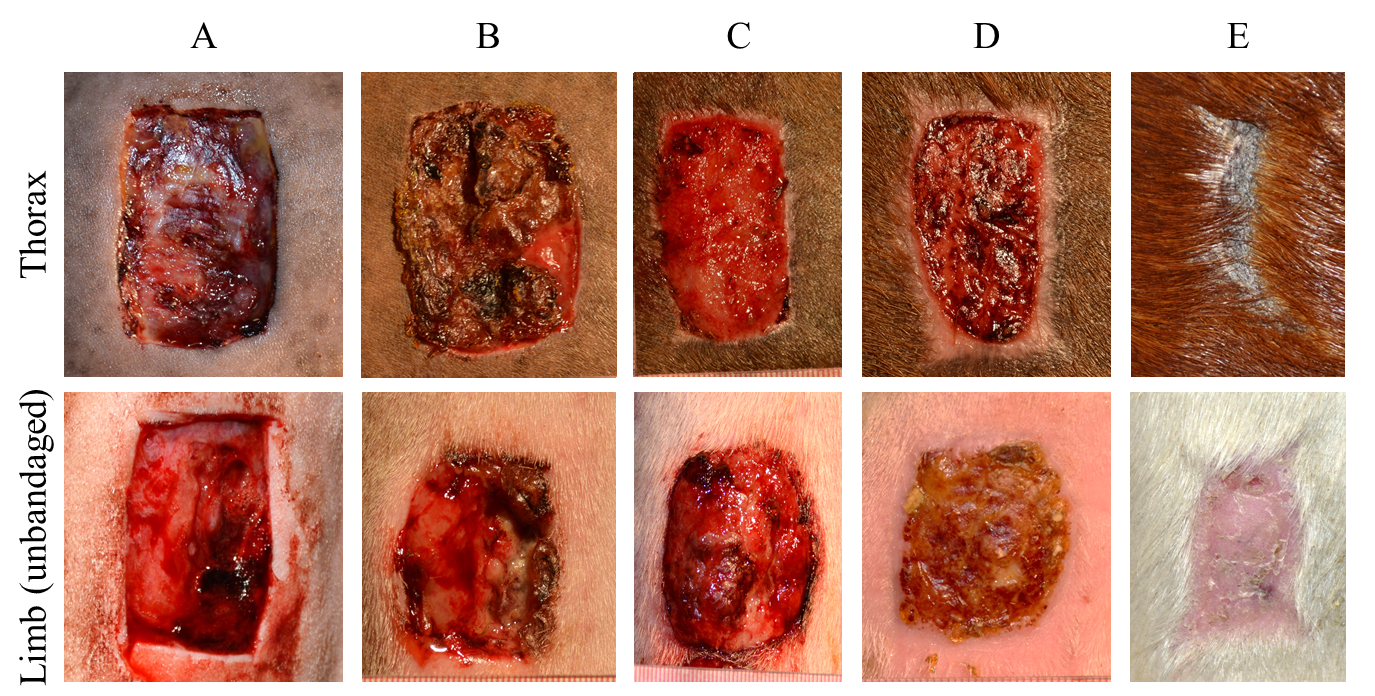

Supplement: Supplementary file 1 — Figure S1. Healing of experimentally induced wounds at the different body sites in a representative horse. A: 24 h, B: 7 days, C: 14 days, D: 21 days, E: cicatrix. (TIF 2334 kb) [file 12917_2019_1829_MOESM1_ESM.tif]
